# Effects of Different Drying Methods on Physicochemical Properties and Nutritional Quality of Abalone Bioactive Peptides

**DOI:** 10.3390/molecules30071516

**Published:** 2025-03-28

**Authors:** Qiting Li, Longxiang Li, Pufu Lai, Yingying Wei, Chunmei Lai, Yusha Liu, Mengjie Yang, Shaoxiong Zhou, Junchen Chen, Junzheng Sun

**Affiliations:** 1 College of Food Science, Fujian Agriculture and Forestry University, Fuzhou 350002, China; lqt6868@163.com (Q.L.); wyy787459024@163.com (Y.W.); yy613666@163.com (Y.L.); yummy2023yummy@163.com (M.Y.); zhousx2023@163.com (S.Z.); 2 Institute of Food Science and Technology, Fujian Academy of Agricultural Sciences, Fuzhou 350003, China; 13616901013@163.com (L.L.); 19911583175@163.com (C.L.); junchenccc@163.com (J.C.); 3 National R & D Center for Edible Fungi Processing, Fuzhou 350003, China; 4 Key Laboratory of Subtropical Characteristic Fruits, Vegetables and Edible Fungi Processing (Co-Construction by Ministry and Province), Ministry of Agriculture and Rural Affairs, Fuzhou 350003, China; 5 College of Life Sciences, Fujian Agriculture and Forestry University, Fuzhou 350002, China

**Keywords:** bioactive peptide, protein, dried, physical and chemical properties, nutritional components

## Abstract

This study conducted a systematic comparison of four drying methods (vacuum freeze-drying, spray drying, spray freeze-drying, and hot air drying) on abalone bioactive peptides, investigating their effects on physicochemical properties and nutritional composition. Scanning electron microscopy revealed distinct morphological characteristics: hot-air-dried samples showed compact structures with large particles, and vacuum-freeze-dried samples exhibited flaky morphology, while spray-freeze-dried and spray-dried samples demonstrated advantageous smaller particle sizes. Spray freeze-drying achieved superior emulsification capacity and fat absorption, significantly higher than hot air drying. The enhanced performance was attributed to increased exposure of hydrophobic amino acid residues and improved surface activity. Regarding nutritional composition, vacuum freeze-drying demonstrated optimal protein and total amino acid preservation, while spray freeze-drying showed the highest retention of Ca and Fe. Interestingly, hot air drying exhibited superior vitamin A retention, attributed to its fat-soluble nature and stability below 100 °C. The particle size reduction in spray-freeze-dried samples enhanced solvent–solute contact area, contributing to improved solubility and consequently superior foaming properties. These findings provide valuable insights into the relationship between drying methods and product characteristics, offering guidance for optimizing processing conditions in marine protein production.

## 1. Introduction

The Abalone (*Haliotis* spp.) is a highly valued marine gastropod renowned for its exceptional nutritional profile, including high-quality proteins, essential amino acids, and bioactive compounds, as well as its distinct organoleptic properties. Traditional Chinese medicine has documented its therapeutic applications for over 2000 years, with beneficial effects on respiratory and digestive systems, as well as tumor-inhibitory, diuretic, and immunoregulatory properties [1,2,3]. Abalone offal is of considerable significance owing to its substantial content of proteins and polysaccharides. Bioactive peptides derived from abalone meat and offal have demonstrated multiple biological activities, including anti-cancer, antioxidant, anti-hypertensive, anti-allergic, anti-inflammatory, and immunomodulatory properties [4,5,6,7,8]. While extensive research has been conducted on the extraction, characterization, and functional properties of abalone bioactive peptides, the impact of different drying methods on their physicochemical properties and nutritional value remains largely unexplored [9,10,11].

Drying is a critical preservation process that reduces water activity to inhibit microbial growth while facilitating storage and transportation, though the process may affect the biological activity of sensitive compounds. The increasing consumer demand for dried products has stimulated intensive research into various drying technologies. Modern industrial drying methods, including hot air drying, spray drying, vacuum freeze-drying, and spray freeze-drying, each possess distinct operational characteristics and effects on product quality [12,13,14,15]. Previous studies have demonstrated that different drying methods can significantly influence not only the nutritional composition but also the structural integrity and biological activity of protein-based materials. For instance, Shi et al. demonstrated that the application of hot air drying and vacuum drying techniques had disparate effects on the nutritional composition of dragon fruit floral parts [16]. While Stamenkovic et al. highlighted the potential nutrient degradation associated with high-temperature hot air drying [17].

The process of dry preservation of peptides, such as freeze-drying, has been shown to significantly inhibit the hydrolysis of peptide bonds, oxidation reactions, and chemical modification of amino acid side chains (such as cysteine and methionine) by eliminating water. In addition, the dehydrated environment can block microbial proliferation, thereby improving stability and extending shelf life [18]. Freeze-dried powder can avoid accumulation/precipitation caused by hydrophobic action or charge change in the liquid and avoid mechanical damage caused by repeated freeze/thaw. Dried peptides can be stored stably at −20 °C for several years, offering both space efficiency and transportation convenience, thereby significantly reducing storage costs. However, it is important to note that the dry powder is susceptible to moisture absorption and must be strictly sealed to prevent degradation. Improper operation during resolution, such as the selection of an inappropriate solvent or excessive oscillation, can result in the destruction of the active structure. Furthermore, peptides containing complex modifications or disulfide bonds necessitate liquid storage at low temperatures (−80 °C) to preserve their conformation. Conversely, the liquid method relies on ultra-low temperatures and additives, a process that is costly and susceptible to interference [19]. Conversely, drying technology has emerged as a prevalent method for peptide preservation, offering long-term stability under simple conditions such as room temperature and the avoidance of light.

The selection of appropriate drying technology for abalone bioactive peptides is crucial for optimizing processing efficiency, maintaining biological activity, and ensuring cost effectiveness in industrial production. This is particularly relevant given the growing interest in their potential applications in functional foods and traditional Chinese medicine formulations [20,21,22,23,24,25]. Furthermore, understanding the synergistic interactions between these peptides and other bioactive compounds presents an emerging research direction with significant implications for their therapeutic applications. This study systematically evaluates and compares the effects of four drying techniques—hot air drying, spray freeze-drying, spray drying, and vacuum freeze-drying—on the physicochemical properties, structural characteristics, and nutritional quality of abalone bioactive peptides, with particular emphasis on protein stability and biological activity retention. The findings will provide crucial insights for optimizing industrial processing parameters and establishing a theoretical framework for the development of functional food and pharmaceutical applications.

## 2. Results

### 2.1. Effects of Different Drying Methods on Physicochemical Properties of Abalone Bioactive Peptides

#### 2.1.1. Solubility

**Figure 1:**
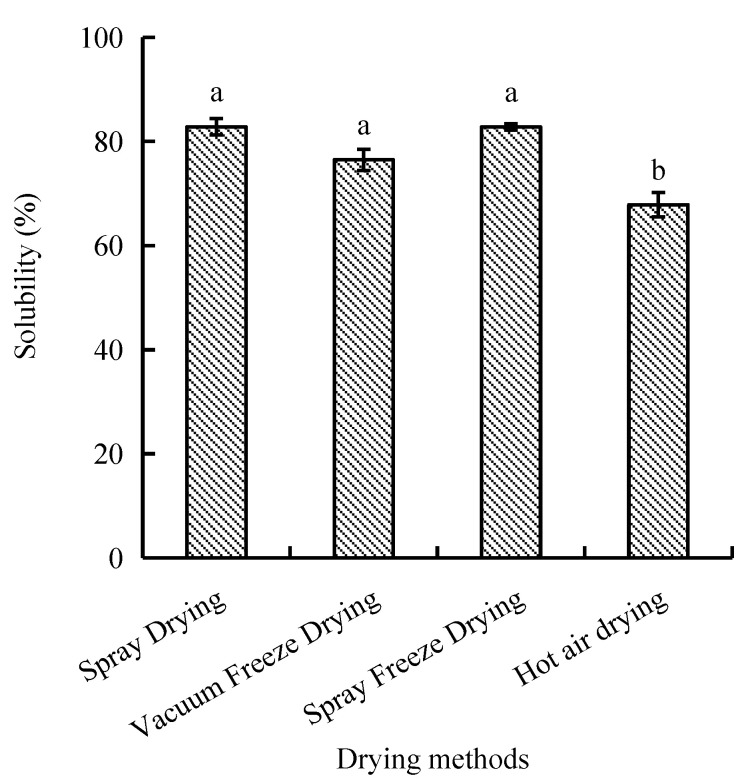
Effect of drying methods on solubility of abalone bioactive peptides. Values are presented as mean ± SD (n = 3). Different letters indicate significant differences (*p* < 0.05).

The protein solubility, defined as the protein’s ability to dissolve in an aqueous solution, differed significantly between hot air drying and the other three drying methods. Spray-dried and spray-freeze-dried samples exhibited the highest solubility values of 82.81% and 82.77%, respectively, with no significant difference between them. In contrast, hot-air-dried samples showed significantly lower solubility (67.83%, *p* < 0.05) compared to other drying methods (Figure 1).

#### 2.1.2. Fat Absorption Capacity

**Figure 2:**
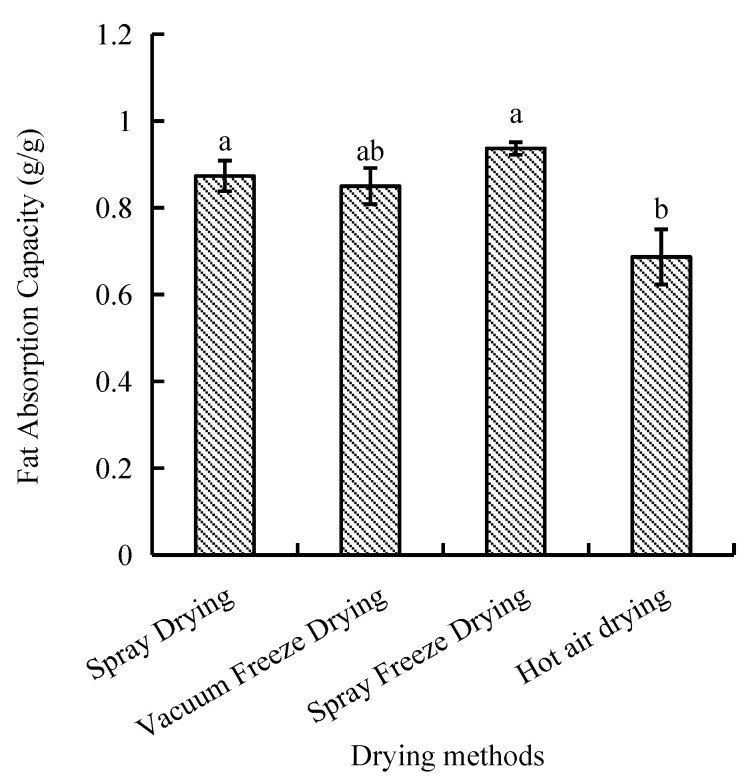
Fat absorption capacity of abalone bioactive peptides processed by different drying methods. Values are presented as mean ± SD (n = 3). Different letters indicate significant differences (*p* < 0.05).

The capacity of fat absorption is a significant functional characteristic that must be taken into account when assessing lipid binding capacity. Among the four drying methods, the fat absorption capacity of samples dried by hot air was the least efficient, showing significant differences compared with spray drying and spray freeze-drying. However, a statistically insignificant difference was observed between the processes of spray drying, vacuum freeze-drying, and spray freeze-drying. The spray-freeze-dried samples demonstrated superior fat absorption capacity (0.94 g/g), while hot-air-dried samples exhibited the lowest value (0.69 g/g, *p* < 0.05) (Figure 2).

#### 2.1.3. Emulsifying Properties

**Figure 3:**
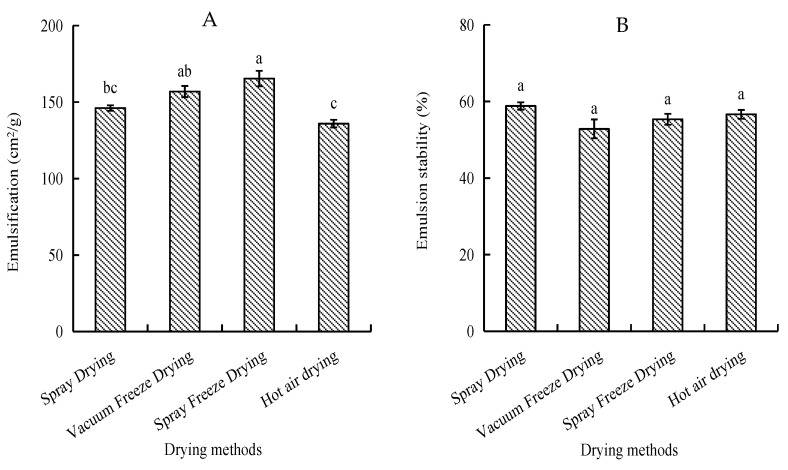
Emulsifying properties of abalone bioactive peptides as influenced by different drying methods: (**A**) emulsifying activity index (EAI); (**B**) emulsion stability index (ESI). Values are presented as mean ± SD (n = 3). Different letters indicate significant differences (*p* < 0.05).

Emulsifying activity index (EAI) and emulsion stability index (ESI) were evaluated to assess the peptides’ ability to form and stabilize oil-in-water emulsions. Spray-freeze-dried samples exhibited the highest EAI (165.32 cm^2^/g), significantly higher than spray-dried and hot-air-dried samples (*p* < 0.05). Hot-air-dried samples showed the lowest EAI (135.88 cm^2^/g). Regarding emulsion stability, spray-dried samples demonstrated the highest ESI (58.81%), while vacuum-freeze-dried samples showed the lowest (52.83%). However, the differences in ESI among all drying methods were not statistically significant (*p* > 0.05) (Figure 3).

#### 2.1.4. Foaming Properties

**Figure 4:**
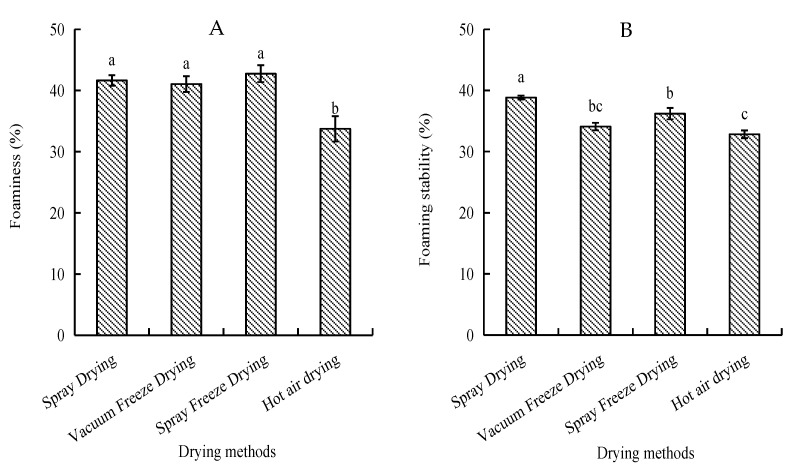
Foaming properties of abalone bioactive peptides as influenced by different drying methods: (**A**) foaming capacity; (**B**) foam stability. Values are presented as mean ± SD (n = 3). Different letters indicate significant differences (*p* < 0.05).

Foam capacity and stability were assessed as indicators of surface-active properties. Spray-freeze-dried samples exhibited the highest foam capacity (42.75%), followed by spray-dried and vacuum-freeze-dried samples, with no significant differences among these methods. Spray-dried samples showed superior foam stability (38.85%), while hot-air-dried samples demonstrated the lowest values for both parameters (Figure 4).

#### 2.1.5. Ultrastructural Characteristics

**Figure 5:**
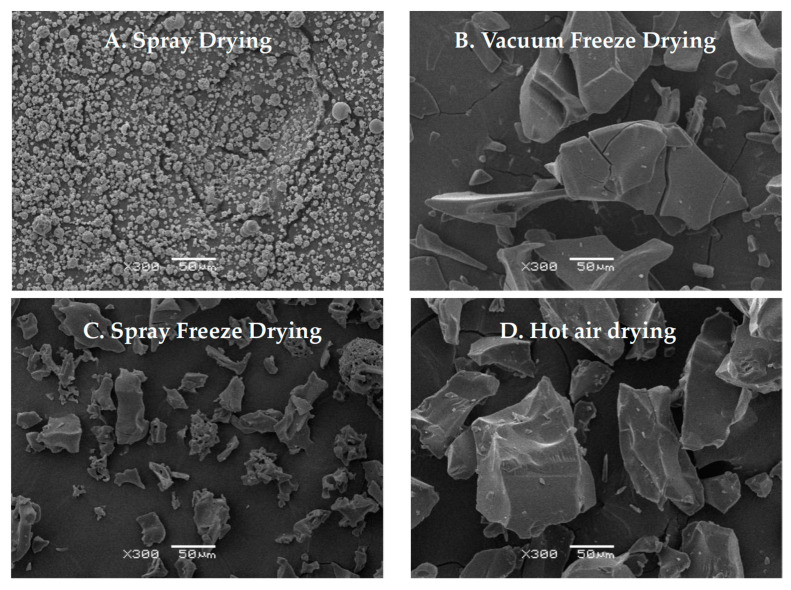
Scanning electron micrographs of abalone bioactive peptides processed by different drying methods: (**A**) hot air drying; (**B**) vacuum freeze-drying; (**C**) spray drying; (**D**) spray freeze-drying.

Scanning electron microscopy revealed distinct morphological characteristics for each drying method (Figure 5). Spray-dried samples showed predominantly spherical particles with uniform size distribution and slightly rough surfaces [26]. Vacuum-freeze-dried samples displayed a characteristic flake-like morphology with relatively smooth surfaces and distinct fissures, consistent with typical freeze-dried material structures. Spray-freeze-dried samples exhibited a distinct porous structure with numerous voids, attributable to the sublimation of ice crystals during the drying process [27]. Hot-air-dried samples exhibited irregular particles with uneven surfaces, minimal porosity, and occasional surface folds. This morphology likely results from prolonged exposure to temperature and humidity gradients, leading to surface tension effects and moisture-migration-induced structural changes [28]. Hot air drying induces material deformation and contraction, while vacuum freeze-drying preserves the material’s original structure through rapid sublimation, albeit with some hydrogen bond disruption [29]. The observed structural differences can be attributed to the unique drying mechanisms of each method.

### 2.2. Effects of Different Drying Methods on the Nutrient Composition of Abalone Bioactive Peptides

#### 2.2.1. Protein Content

**Figure 6:**
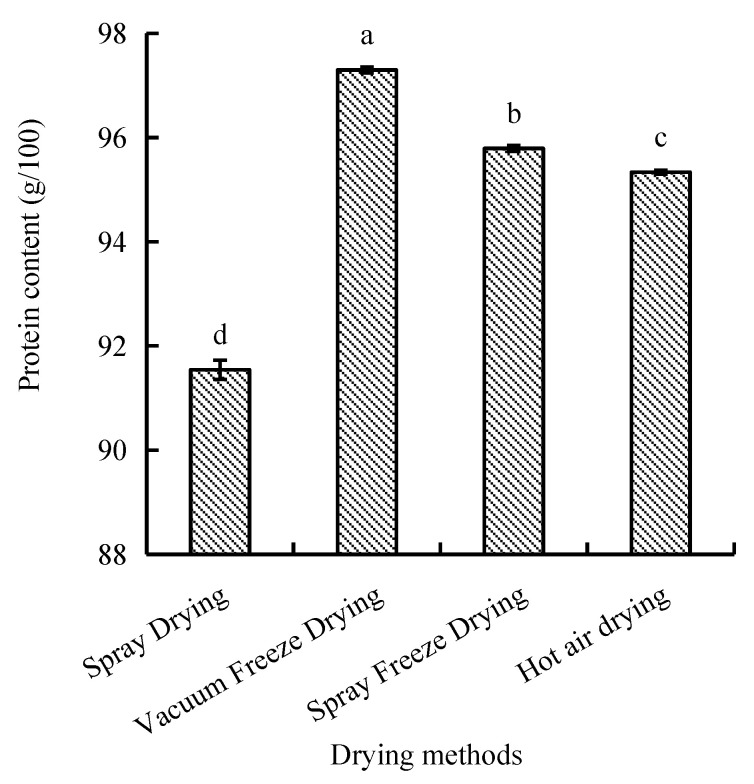
Protein content of abalone bioactive peptides processed by different drying methods. Values are presented as mean ± SD (n = 3). Different letters indicate significant differences (*p* < 0.05).

The protein content of abalone bioactive peptides varied significantly among different drying methods, ranging from 91.5 to 97.3 g/100 g. Vacuum-freeze-dried samples exhibited the highest protein content, followed by spray-freeze-dried, hot-air-dried, and spray-dried samples, with significant differences observed between methods (*p* < 0.05) (Figure 6).

#### 2.2.2. Amino Acid Content

Amino acid analysis revealed the presence of 17 amino acids, with total content ranging from 85.69 to 91.30 g/100 g across different drying methods. Glycine, glutamic acid, aspartic acid, arginine, alanine, and proline were predominant, while cystine showed the lowest concentration. Vacuum freeze-drying preserved the highest total amino acid content, significantly higher than spray drying (*p* < 0.05). Spray freeze-drying and hot air drying showed intermediate values with no significant differences between them (Table 1).

#### 2.2.3. Ash Content

**Figure 7:**
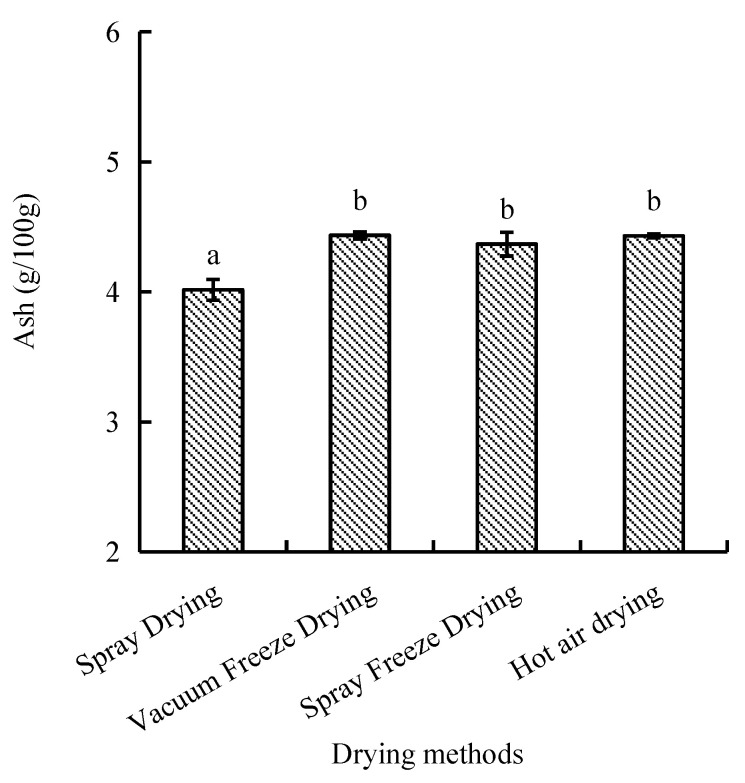
Ash content of abalone bioactive peptides processed by different drying methods. Values are presented as mean ± SD (n = 3). Different letters indicate significant differences (*p* < 0.05).

Ash content, representing inorganic constituents, ranged from 4.0 to 4.4 g/100 g across drying methods. Vacuum-freeze-dried and hot-air-dried samples showed comparable ash contents, while spray-dried samples exhibited significantly lower values (*p* < 0.05). Spray-freeze-dried samples demonstrated intermediate ash content (Figure 7).

#### 2.2.4. Mineral Content

**Figure 8:**
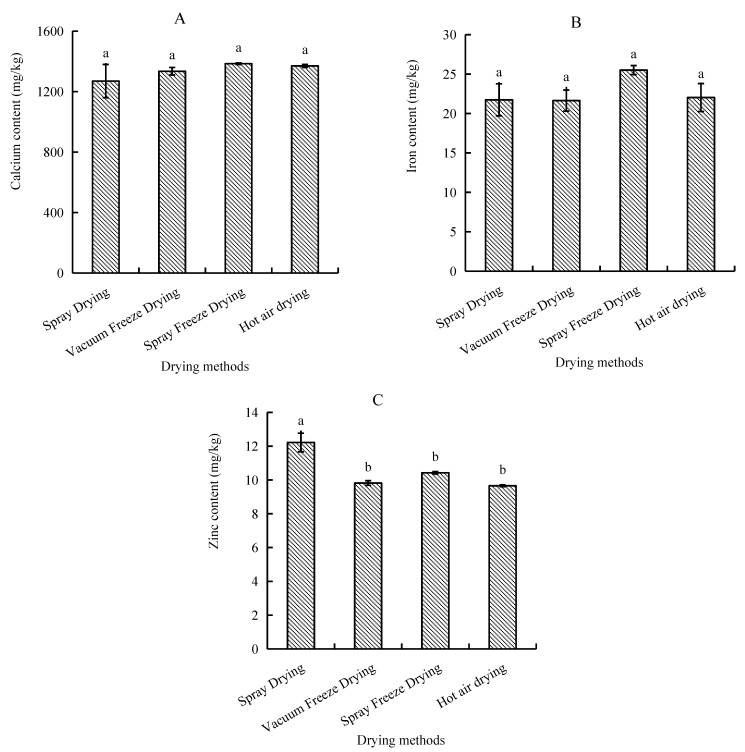
Mineral content of abalone bioactive peptides as influenced by different drying methods: (**A**) calcium; (**B**) iron; (**C**) zinc. Values are presented as mean ± SD (n = 3). Different letters indicate significant differences (*p* < 0.05).

Analysis of mineral composition revealed distinct patterns among trace elements, with calcium being the predominant mineral, followed by iron and zinc. Samples subjected to spray freeze-drying demonstrated the highest mineral retention, with calcium content reaching 1385.0 mg/kg and iron content at 25.5 mg/kg. In contrast, spray-dried samples exhibited the lowest calcium concentration (1270.0 mg/kg), while vacuum-freeze-dried samples recorded the minimum iron levels (21.6 mg/kg). However, zinc distribution showed a different pattern, with spray-dried samples exhibiting the highest content, followed by spray-freeze-dried, vacuum-freeze-dried, and hot-air-dried samples (Figure 8).

#### 2.2.5. Vitamin A Content

**Figure 9:**
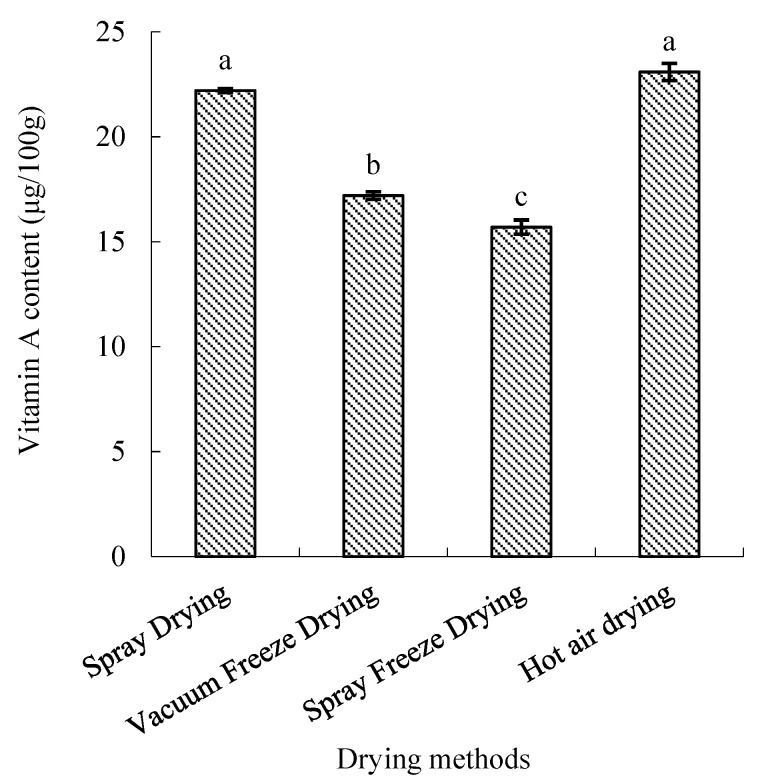
Vitamin A content of abalone bioactive peptides processed by different drying methods. Values are presented as mean ± SD (n = 3). Different letters indicate significant differences (*p* < 0.05).

Vitamin A retention varied significantly among drying methods (*p* < 0.05). Hot-air-dried samples maintained the highest vitamin A content, significantly higher than vacuum-freeze-dried and spray-freeze-dried samples (*p* < 0.05) but comparable to spray-dried samples. The retention efficiency followed the order: hot air drying > spray drying > vacuum freeze-drying > spray freeze-drying (Figure 9).

## 3. Discussion

### 3.1. Impact of Drying Methods on Physicochemical Properties

The selection of appropriate drying methods significantly influences both the structural integrity and functional properties of bioactive peptides. Our comprehensive analysis reveals that the impact of different drying techniques extends beyond simple dehydration, involving complex molecular interactions that affect product quality at multiple levels.

The comparative analysis of four drying methods (vacuum freeze-drying, spray drying, spray freeze-drying, and hot air drying) revealed significant variations in the physicochemical properties of abalone bioactive peptides. SEM analysis revealed that samples after hot air drying exhibited a relatively compact structure with large particles, while samples after vacuum freeze-drying demonstrated a flaky morphology and large particle size. In contrast to the aforementioned drying methods, samples after spray freeze-drying and spray drying showed smaller particle sizes (Figure 5). The solubility of the samples subjected to spray drying and spray freeze-drying was higher than that of the samples exposed to hot air drying and vacuum freeze-drying. The reduction in particle size enhanced the contact area between the solvent and the solute, thereby promoting increased solubility. The relationship between particle morphology and functional properties can be explained through the principles of surface chemistry and molecular interactions. Smaller particles increase the specific surface area available for interaction, leading to enhanced functional properties through multiple mechanisms, including improved water penetration and molecular mobility, reduced diffusion distance for solvent–solute interactions, and enhanced interfacial phenomena.

In comparison with the other three drying methods, spray freeze-drying exhibited the highest emulsification capacity (165.32 cm^2^/g). This phenomenon may be attributed to the exposure of hydrophobic amino acid residues to the water–oil interface during the process of peptide molecule dissociation, unfolding, or conformational changes, thereby augmenting the surface activity of the interface [30]. The comparatively low emulsification of hot air drying (135.88 cm^2^/g) can be attributed to its low solubility (67.83%). Hot-air-dried samples exhibit larger particle sizes and lower solubility, resulting in reduced contact surface area with the solvent and consequently decreased emulsification performance [31].

Good solubility is a fundamental prerequisite for protein foaming [32]. Spray-freeze-dried samples exhibited superior foaming properties, which can be attributed to their enhanced solubility. This relationship between solubility and foaming properties is consistent with previous research, which demonstrated that higher solubility facilitates better foam formation and stability [32]. Furthermore, spray-freeze-dried samples with smaller particle sizes can be adsorbed more quickly during churning or bubbling, resulting in a higher foam capacity than hot-air-dried samples (33.73%) [33].

The fat absorption capacity of the spray-freeze-dried samples (0.94 g/g) was significantly higher than that of the hot-air-dried samples (0.69 g/g, *p* < 0.05). This difference can be attributed to the exposure of hydrophobic amino acids in peptide molecules and the enhancement of surface hydrophobicity [31].

### 3.2. Effects on Nutritional Composition

#### 3.2.1. Protein and Amino Acid Content

Vacuum freeze-drying demonstrated superior preservation of protein and total amino acid content compared to other drying methods, consistent with findings by Xu et al. [34]. The reduction in protein content of the spray-dried abalone peptide samples can be attributed to the thermal degradation of heat-sensitive amino acids, particularly sulfur-containing amino acids (e.g., cysteine, methionine). This mechanism contributes to the decrease in amino acid nitrogen content through the formation of volatile nitrogenous compounds such as ammonia. The lower protein retention in hot-air-dried samples likely results from extended exposure to high temperatures, leading to protein denaturation [34]. The superior protein preservation in vacuum-freeze-dried samples can be attributed to the low-temperature processing environment.

The differential protein retention patterns observed across drying methods can be explained through the kinetics of protein denaturation. The relationship between temperature exposure and protein degradation follows an Arrhenius-type behavior, where even small increases in temperature can significantly accelerate denaturation rates. This explains why methods involving rapid temperature transitions (spray freeze-drying) or consistently low temperatures (vacuum freeze-drying) show superior protein preservation compared to methods with prolonged thermal exposure.

#### 3.2.2. Mineral Content

The differential effects of drying methods on mineral content (Ca, Fe, and Zn) demonstrated method-specific retention patterns. Spray freeze-drying yielded the highest Ca and Fe retention rates, while spray drying showed superior Zn preservation. Previous research has demonstrated that freeze-drying and vacuum drying at 70 °C maintain mineral content comparable to fresh products [35]. The relative stability of mineral content across different drying methods aligns with earlier studies indicating minerals’ general resistance to thermal processing [36]. The superior retention of Ca and Fe in spray-freeze-dried samples may be attributed to the formation of stable protein–mineral complexes during the rapid freezing phase, which helps prevent mineral loss during subsequent processing steps. The enhanced Zn preservation in spray-dried samples suggests that rapid water removal may help maintain the integrity of Zn-binding protein structures. Whereas, significant differences with the other three drying methods indicate that drying time has an effect on Zn retention. The observed variations in mineral retention between different drying methods remained within a narrow range, further supporting the inherent stability of these minerals during processing [37].

#### 3.2.3. Vitamin A Retention

Hot air drying unexpectedly demonstrated superior vitamin A retention compared to other methods. This finding, though counterintuitive given the thermal sensitivity of many vitamins, can be explained by vitamin A’s fat-soluble nature and its stability at temperatures below 100 °C [38]. The hydrophobic characteristics of vitamin A may have contributed to its stability during water removal, while its encapsulation within the food matrix could provide additional protection against thermal degradation, even under elevated drying temperatures. In contrast, other drying methods may expose vitamin A to oxidative stress during processing, potentially leading to greater losses.

### 3.3. Industrial Implications and Future Perspectives

The findings of this study have significant implications for industrial-scale production of bioactive peptides. The superior performance of spray freeze-drying in maintaining both physicochemical properties and nutritional value must be balanced against its higher operational costs compared to conventional drying methods. For high-value products where maintaining biological activity is crucial, spray freeze-drying offers the best compromise between quality and processing efficiency. For large-scale production where cost considerations are paramount, spray drying may provide an acceptable alternative, particularly when coupled with protective additives to minimize thermal degradation.

## 4. Materials and Methods

### 4.1. Materials and Reagents

#### 4.1.1. Materials

The abalone bioactive peptide stock solution was prepared using abalone viscera supplied by Fuzhou Rixing Aquatic Foods Co., Ltd. (Fuzhou, China). The frozen viscera (−18 °C) were homogenized and suspended in NaOH solution (pH 12.0). The mixture was subjected to ultrasonic extraction followed by centrifugation at 10,000 rpm for 10 min. The supernatant was adjusted to pH 4.5 with HCl solution and maintained at room temperature for 30 min to precipitate proteins. After centrifugation (10,000 rpm, 10 min), the precipitate was collected and hydrolyzed using 2.50 µg/g commercial marine proteases (30 min, 50 °C) to obtain the stock solution (molecular weight < 1000 Da).

#### 4.1.2. Reagents

BCA protein content detection kit was purchased from Sigma-Aldrich Trading Co., Ltd. (Shanghai, China). All other analytical grade chemicals including sodium dodecyl sulfate (SDS), copper sulfate, potassium sulfate, sulfuric acid, boric acid, methyl red indicator, bromocresol green indicator, methylene blue indicator, sodium hydroxide, and ethanol were obtained from SINOPHARM CHEMICAL REAGENT CO., Ltd. (Shanghai, China). All solutions were prepared using ultrapure water.

### 4.2. Experimental Methodology

#### 4.2.1. Drying Methods

##### Spray Drying

Spray drying is a method that transforms liquid feed into dried particles by atomizing the feed into a hot drying medium. The process involves four stages: atomization, spray-air contact, drying, and separation. The Yamato ADL311 spray dryer, manufactured in Japan (Yamato, Japan), has a maximum water evaporation capacity of 1300 mL/h. It features an inlet temperature adjustment range of 40−220 °C, an outlet temperature controllable interval of 0−60 °C, and a drying air volume of 0−0.7 m^3^/min with continuous adjustment. The spray gas pressure is adjustable within a range of 0−0.6 MPa.

In this study, the peptide solution was spray-dried using the following parameters: feed solution flow rate of 15 mL/min, compressed air flow rate of 0.6 m^3^/h, inlet temperature of 105 °C, and outlet temperature of 55 °C. Drying was terminated when no powder was observed at the outlet.

##### Vacuum Freeze-Drying

Vacuum freeze-drying is a dehydration process that removes water through sublimation under vacuum conditions. The process consists of three main phases: freezing, primary drying (sublimation), and secondary drying (desorption). The Qingdao Yonghe Chuangxin (Qingdao, China) CTFD-30MD Vacuum Freeze Dryer is a piece of equipment which has been designed for use in the food industry. It has a temperature control range of −50 °C to + 70 °C for the spacer, a no-load temperature of the cold trap of ≤−80 °C, and an ultimate vacuum of <10 Pa. The drying area of the equipment is 0.36 m^2^, and the power consumption of the entire machine is 3.5 kW.

The peptide solution was vacuum freeze-dried through a five-stage process: initial freezing temperature −30 °C, followed by drying at −10 °C for 2 h in the 1st stage, 0 °C for 2 h in the 2nd stage, 20 °C for 3 h in the 3rd stage, 40 °C for 5 h in the 4th stage, and 60 °C for 12 h in the 5th stage, then end the drying and take out the powder.

##### Spray Freeze-Drying

Spray freeze-drying combines the advantages of both spray drying and freeze-drying. The process involves atomizing the solution into a cold chamber for instant freezing, followed by sublimation under vacuum conditions. This method produces highly porous particles with excellent rehydration properties. The spray freeze dryer (YC-3000, Yacheng, Shanghai, China) has a single processing capacity of 2000 mL (drying cycle ≤ 6 h), spray freezing temperature < −15 °C, cold trap temperature control ≤ −60 °C, ultimate vacuum pressure < 20 Pa (no load), spray pressure 2–5 BAR adjustable. The supporting system integrates a high-efficiency vacuum pump (power < 2 KW), an air compressor (air output 4.2 M^3^/h) and a cold air circulation system (air volume 5.5 M^3^/min).

The peptide solution was spray freeze-dried with an initial spraying temperature of −30 °C. The process was terminated and the powder was collected when the material temperature reached 45 °C and the chamber vacuum pressure stabilized at 1–5 Pa.

##### Hot Air Drying

Hot air drying is a conventional drying method that uses heated air to remove moisture through convection and conduction heat transfer. The electric blast drying oven (101A, Rongji Da, Beijing, China) used in this study operates within a temperature range of +10 to 250 °C. The equipment has a chamber volume of 43 L, and heating power of 1200 W.

The peptide solution was hot air dried at 60 °C for 48 h.

#### 4.2.2. Physicochemical Analysis

##### Solubility Determination

Solubility was determined according to Mokni Ghribi et al. with minor modifications [39]. Dried peptide powder (0.5 g) was dissolved in distilled water (49.5 mL) and stirred continuously at room temperature (25 ± 2 °C) for 30 min. The solution was centrifuged (3500 rpm, 15 min, 20 °C), and protein content in the supernatant was quantified using the BCA protein content assay kit. The analysis was performed in triplicate. Solubility was calculated as follows:

(1) $$\mathrm{Solubility}\;(\%)=\frac{M}{M_{A}}\times 100\%$$

where M is the protein content in the supernatant (g), and M_A_ is the total protein content of the sample (g).

##### Fat Absorption Capacity

Fat absorption capacity was determined following the method of Miah et al. [40]. Soybean oil (10.0 g) was gradually added to dried peptide powder (1.0 g) and thoroughly mixed. After standing for 30 min at room temperature (25 ± 2 °C), the mixture was centrifuged at 6000 rpm for 12 min. The supernatant oil was carefully aspirated and weighed. The analysis was performed in triplicate. Fat absorption capacity was calculated as follows:

(2) Fat absorption capacity (g/g)=10−M

where M is the mass of supernatant oil (g).

##### Emulsifying Properties

Emulsifying properties were determined according to Taheri et al. [41]. Dried peptide powder (1.0 g) was dissolved in distilled water (99 mL). An aliquot (24 mL) of this solution was combined with soybean oil (8 mL) and homogenized at 10,000 rpm for 1 min. The homogenized solution (1 mL) was diluted with 0.1% SDS solution (49 mL). Absorbance was measured at 500 nm using a microplate reader, with 0.1% SDS solution as blank. The analysis was performed in triplicate. Emulsifying activity index (EAI) was calculated as follows:

(3) $$\mathrm{Emulsifiability}\left({\mathrm{cm}}^{2}/g\right)=\frac{2.3\times 2\times A}{m\times K}$$

where A is the OD value at 500 nm; m is the mass of peptide powder (g); and K = 0.25 (oil volume fraction).

Emulsion stability was determined by measuring the absorbance after 10 min standing and calculated as follows:

(4) $$\mathrm{Emulsion}\;\mathrm{stability}\;\left(\%\right)=\frac{A\times 10}{A-A_{10}}$$

where A is the OD value at 500 nm of the solution; and A_10_ is the OD value at 500 nm after 10 min of standing.

##### Foaming Properties

Foaming properties were determined following Hammershøj et al. [42]. Peptide powder was dissolved in distilled water to prepare a 1.0% (*w*/*v*) solution. The solution (20 mL) was rapidly stirred for 3 min. Foam volume was recorded immediately (V_1_) and after 30 min standing (V_2_). The analysis was performed in triplicate. Foam capacity and stability were calculated as follows:

(5) $$\mathrm{Foam}\;\mathrm{capacity}\;\left(\%\right)=\frac{V_{1}}{20}\times 100\%$$

(6) $$\mathrm{Foam}\;\mathrm{stability}\;\left(\%\right)=\frac{V_{2}}{V_{1}}\times 100\%$$

where V_1_ is volume of foam after stirring for 3 min (mL); and V_2_ is volume of foam after standing for 30 min (mL).

##### Ultrastructure Analysis

The morphology of dried peptide powders was examined using a scanning electron microscope (JSM-6380lv, JEOL, Tokyo, Japan). Samples were mounted on aluminum stubs using double-sided carbon tape and sputter-coated with gold. Images were captured at 300× magnification.

#### 4.2.3. Measurement of Nutritional Indicators

##### Protein Content

Protein content was determined using the BCA protein content assay kit according to the manufacturer’s instructions.

##### Amino Acid Content

Amino acid composition was analyzed according to GB 5009.124-2016 (China National Food Safety Standard) [43]. Briefly, samples (100 mg) were hydrolyzed with 6 M HCl in vacuum-sealed tubes under nitrogen atmosphere at 110 °C for 22 h. After hydrolysis, the samples were filtered and evaporated to dryness under vacuum using a rotary evaporator. The dried residues were reconstituted in sodium citrate buffer (pH 2.2) and filtered through 0.22 μm membrane filters prior to analysis. The analysis was performed in triplicate using an amino acid analyzer, and amino acid content was calculated using the following equation:

(7) $$X_{i}=\frac{c_{i}\times F\times V\times M}{m\times 10^{9}}\times 100$$

where $c_{i}$ is the amino acid content in the sample (nmol/mL); F is the dilution; V is the constant volume of the sample hydrolysate (mL); M is the molar mass of amino acid (g/mol); and m is the sample mass (g).

##### Ash Content

Ash content was determined according to GB 5009.4-2016 (China National Food Safety Standard) [44]. Briefly, samples (5.0 g) were moistened with 1 mL magnesium acetate solution and dried in a boiling water bath. The samples were then charred on a hot plate until smoking ceased, followed by incineration in a muffle furnace at 550 °C for 4 h. The samples were cooled to approximately 200 °C and transferred to a desiccator for 30 min. The incineration process was repeated until the difference between consecutive weighings was less than 0.5 mg. The analysis was performed in triplicate, and ash content was calculated using the following equation:

(8) $$X=\frac{m_{1}-m_{2}-m_{0}}{m_{3}-m_{2}}\times 100$$

where $m_{1}$ is the crucible and the ash mass (g); $m_{2}$ is the crucible mass (g); $m_{0}$ is magnesium oxide (magnesium acetate after burning) mass (g); and $m_{3}$ is the crucible and sample mass (g).

##### Mineral Content

(1) Calcium content

Calcium content was determined according to GB 5009.92-2016 (China National Food Safety Standard) [45]. Briefly, samples (1.0 g) were digested with 10 mL nitric acid and 0.5 mL perchloric acid on an electric heating plate until the solution became light yellow or colorless. After cooling, the digested solution was diluted to volume with deionized water, and lanthanum solution was added as a releasing agent. Reagent blanks were prepared following the same procedure. The absorbance was measured at 422.7 nm using atomic absorption spectrometry. The analysis was performed in triplicate, and calcium content was calculated using the following equation:

(9) $$X=\frac{\left(\rho -\rho_{0}\right)\times V\times f}{m}$$

where ρ is the mass concentration of the sample calcium (mg/L); $\rho_{0}$ is the mass concentration of calcium in the blank solution (mg/L); *f* is the dilution of the sample (mg/L); V is the constant volume of the sample (mL); and m is the sample mass (g).

(2) Iron content

Iron content was determined according to GB 5009.90-2016 (China National Food Safety Standard) [46]. Briefly, samples (1.0 g) were digested with 10 mL nitric acid and 0.5 mL perchloric acid on an electric heating plate until the solution became light yellow or colorless. After cooling, the digested solution was diluted to volume with deionized water. Reagent blanks were prepared following the same procedure. The absorbance was measured at 248.3 nm using atomic absorption spectrometry. The analysis was performed in triplicate, and iron content was calculated using the following equation:

(10) $$X=\frac{\left(\rho -\rho_{0}\right)\times V}{m}$$

where ρ is the mass concentration of the sample iron (mg/L); $\rho_{0}$ is the mass concentration of iron in the blank solution (mg/L); V is the constant volume of the sample (mL); and m is the sample mass (g).

(3) Zinc content

Zinc content was determined according to GB 5009.14-2017 (China National Food Safety Standard) [47]. Briefly, samples (1.0 g) were digested with 10 mL nitric acid and 0.5 mL perchloric acid on an electric heating plate. After digestion, the samples were cooled to room temperature and diluted with deionized water. Reagent blanks were prepared following the same procedure. The absorbance was measured at 213.9 nm using atomic absorption spectrometry. The analysis was performed in triplicate, and zinc content was calculated using the following equation:

(11) $$X=\frac{\left(\rho -\rho_{0}\right)\times V}{m}$$

where ρ is the mass concentration of the sample zinc (mg/L); $\rho_{0}$ is the mass concentration of zinc in the blank solution (mg/L); V is the constant volume of the sample (mL); and m is the sample mass (g).

##### Vitamin A Content

Vitamin A content was determined according to GB 5009.82-2016 (China National Food Safety Standard) [48]. Samples (2.0 g) were saponified with potassium hydroxide solution, followed by extraction with n-hexane. The extract was washed with deionized water until neutral, dehydrated with anhydrous sodium sulfate, and concentrated under nitrogen. The concentrate was reconstituted with mobile phase and filtered through 0.22 μm membrane filters prior to analysis. The analysis was performed using high-performance liquid chromatography (HPLC) in triplicate, and vitamin A content was calculated using the following equation:

(12) $$X=\frac{\rho \times V\times 100}{m}$$

where ρ is the vitamin concentration (μg/mL) in the sample calculated by the standard curve; V is the constant volume (mL); and m is the weighing sample (g).

#### 4.2.4. Statistical Analysis

All experiments were performed in triplicate, and values are presented as mean ± SD (n = 3). Statistical analysis was performed using IBM SPSS 22.0 software. Different superscript letters indicate significant differences (*p* < 0.05).

## 5. Conclusions

This comprehensive investigation into four industrial drying methods (spray freeze-drying, hot air drying, vacuum freeze-drying, and spray drying) revealed significant differences in their impact on abalone bioactive peptides’ quality attributes. Spray freeze-drying demonstrated superior performance in maintaining physicochemical properties, achieving optimal emulsification capacity, enhanced solubility, and improved fat absorption, while producing uniform particle morphology that contributed to superior functional characteristics. Although vacuum freeze-drying showed advantages in protein retention and spray drying offered operational efficiency, the unexpected finding of superior vitamin A retention in hot-air-dried samples highlighted the complex nature of nutrient stability during processing. These results establish a clear process–structure–function relationship in dried abalone peptides, with each method presenting distinct advantages for specific quality parameters.

The findings of this study provide crucial insights for industrial applications, suggesting that while spray freeze-drying emerges as the optimal method for high-value products, the selection of drying technology should balance product quality requirements with processing costs and intended applications. Future research should address the optimization of processing parameters, long-term stability of dried products, and cost-effective scaling solutions to advance the commercial production of high-quality bioactive peptides. These insights contribute to the fundamental understanding of drying technology effects on marine bioactive compounds and offer practical guidelines for industrial implementation.

## Figures and Tables

**Table 1:** Amino acid composition of abalone bioactive peptides as influenced by different drying methods. Values are expressed in g/100 g dry weight basis (mean ± SD, n = 3). Different letters indicate significant differences (*p* < 0.05).

Amino Acid Type	Spray Drying	Vacuum Freeze-Drying	Spray Freeze-Drying	Hot Air Drying
Aspartic acid	8.10 ± 0.03 ^b^	8.59 ± 0.24 ^a^	8.48 ± 0.15 ^ab^	8.38 ± 0.12 ^ab^
Threonine	3.65 ± 0.00 ^b^	3.87 ± 0.09 ^a^	3.84 ± 0.06 ^a^	3.78 ± 0.06 ^ab^
Serine	4.38 ± 0.01 ^b^	4.66 ± 0.10 ^a^	4.60 ± 0.05 ^a^	4.50 ± 0.09 ^ab^
Glutamic acid	13.00 ± 0.02 ^b^	13.74 ± 0.30 ^a^	13.66 ± 0.17 ^a^	13.52 ± 0.29 ^ab^
Glycine	13.24 ± 0.06 ^a^	14.02 ± 0.41 ^a^	13.90 ± 0.26 ^a^	13.88 ± 0.28 ^a^
Alanine	7.11 ± 0.00 ^b^	7.55 ± 0.18 ^a^	7.47 ± 0.10 ^ab^	7.38 ± 0.21 ^ab^
Cystine	0.28 ± 0.01 ^b^	0.37 ± 0.01 ^a^	0.32 ± 0.01 ^ab^	0.27 ± 0.03 ^b^
Valerenine	3.06 ± 0.01 ^b^	3.24 ± 0.07 ^a^	3.19 ± 0.04 ^ab^	3.14 ± 0.07 ^ab^
Methionine	1.91 ± 0.00 ^a^	2.11 ± 0.06 ^a^	2.00 ± 0.04 ^a^	1.95 ± 0.12 ^a^
Isoleucine	2.40 ± 0.03 ^c^	2.57 ± 0.03 ^a^	2.50 ± 0.00 ^ab^	2.45 ± 0.04 ^bc^
Leucine	4.45 ± 0.05 ^b^	4.80 ± 0.17 ^a^	4.65 ± 0.12 ^ab^	4.65 ± 0.05 ^ab^
Tyrosine	1.66 ± 0.02 ^c^	1.90 ± 0.05 ^a^	1.74 ± 0.04 ^bc^	1.79 ± 0.03 ^b^
Phenylalanine	2.16 ± 0.00 ^c^	2.35 ± 0.04 ^a^	2.27 ± 0.03 ^bc^	2.24 ± 0.02 ^b^
Lysine	4.68 ± 0.01 ^b^	5.01 ± 0.11 ^a^	4.89 ± 0.06 ^ab^	4.74 ± 0.12 ^b^
Histidine	1.14 ± 0.02 ^b^	1.23 ± 0.02 ^a^	1.18 ± 0.00 ^ab^	1.11 ± 0.04 ^b^
Arginine	7.82 ± 0.01 ^b^	8.37 ± 0.20 ^a^	8.13 ± 0.10 ^ab^	8.16 ± 0.22 ^ab^
Proline	6.68 ± 0.14 ^b^	6.93 ± 0.02 ^a^	6.96 ± 0.05 ^a^	6.88 ± 0.08 ^ab^
Total Amino Acid	85.69 ± 0.08 ^b^	91.30 ± 2.08 ^a^	89.77 ± 1.16 ^ab^	88.84 ± 1.62 ^ab^

Different letters in the same row indicate significant differences between different drying methods (*p* < 0.05).

## Data Availability

The original contributions presented in the study are included in the article. Further inquiries can be directed to the corresponding author.
